# Zr- and Ce-doped Li_6_Y(BO_3_)_3_ electrolyte for all-solid-state lithium-ion battery†

**DOI:** 10.1039/d1ra02191e

**Published:** 2021-05-05

**Authors:** Toyoki Okumura, Yoshitaka Shiba, Noriko Sakamoto, Takeshi Kobayashi, Saori Hashimoto, Kentaro Doguchi, Harunobu Ogaki, Tomonari Takeuchi, Hironori Kobayashi

**Affiliations:** Research Institute of Electrochemical Energy, National Institute of Advanced Industrial Science and Technology (AIST) Midorigaoka 1-8-31, Ikeda Osaka 563-8577 Japan toyoki-okumura@aist.go.jp; Development Division, Canon Optron Inc. Kanakubo1744-1, Yuki Ibaraki 307-0015 Japan

## Abstract

The ionic conductivity of Li_6_Y(BO_3_)_3_ (LYBO) was enhanced by the substitution of tetravalent ions (Zr^4+^ and Ce^4+^) for Y^3+^ sites through the formation of vacancies at the Li sites, an increase in compact densification, and an increase in the Li^+^-ion conduction pathways in the LYBO phase. As a result, the ionic conductivity of Li_5.875_Y_0.875_Zr_0.1_Ce_0.025_(BO_3_)_3_ (ZC-LYBO) reached 1.7 × 10^−5^ S cm^−1^ at 27 °C, which was about 5 orders of magnitude higher than that of undoped Li_6_Y(BO_3_)_3_. ZC-LYBO possessed a large electrochemical window and was thermally stable after cosintering with a LiNi_1/3_Mn_1/3_Co_1/3_O_2_ (NMC) positive electrode. These characteristics facilitated good reversible capacities in all-solid-state batteries for both NMC positive electrodes and graphite negative electrodes *via* a simple cosintering process.

## Abstract 2

Ionic conductivity of Li_6_Y(BO_3_)_3_ (LYBO) is enhanced by the substitution of tetravalent ions through an increase in the conduction pathways *etc.* Zr,Ce-doped LYBO can be used as an electrolyte for all-solid-state batteries *via* a cosintering process.

## Graphical abstract

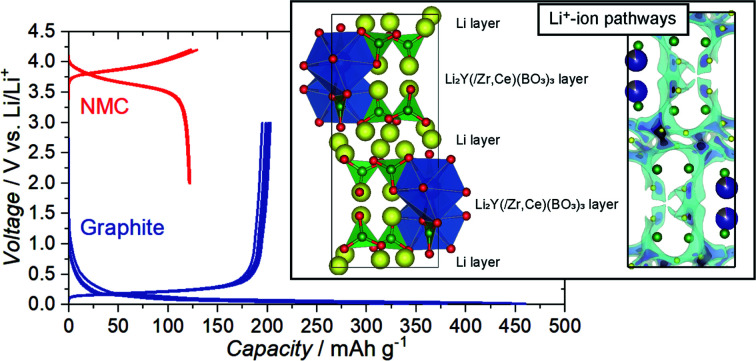

## Introduction

The steady evolution of new crystal systems with high ionic conductivities has recently heightened expectations for the realisation of all-solid-state batteries (ASSBs).^1^ Indeed, the successful demonstration of a high-rate-capacity ASSB has been reported with 10^−2^ S cm^−1^-class Li_10_GeP_2_S_12_ (LGPS)-type sulfide-based electrolytes.^2^ In the case of oxides, the reported conductivities of ≈10^−3^ S cm^−1^ in current oxide-based crystal systems (perovskites,^3^ NASICON types,^4^ and garnets^5^) have encouraged the realisation of oxide-based ASSBs because they are approaching the conductivities of non-aqueous liquid electrolytes (≈10^−2^ S cm^−1^). Oxide-based ASSBs are expected to be applied as on-board power sources for next-generation wireless Internet-of-Things devices because of their long-life performance and thermal stability. However, the discovery of better ion-conductive oxides is not always related to the enhancement of the performance of oxide-based ASSBs. This corresponds to another issue, that is, the difficulty of impurity-free interface formation between the active materials and oxide electrolyte during cosintering.^6–10^ Therefore, oxide-based ionic conductors that are both highly conductive and thermally stable with respect to the electrode materials are required for the development of electrolytes for sintered oxide-based ASSBs.

The Li_3_BO_3_-related families have been considered as suitable electrolytes for fabricating good contacts at the electrode/electrolyte interface by cosintering. For example, Li_3_BO_3_ was used as an interfacial additive between LiCoO_2_ electrode particles and a garnet-type sintered electrolyte plate, and reversible charge–discharge capacities were demonstrated for the ASSB after cosintering at 700 °C, despite the low conductivity of Li_3_BO_3_ (*σ* < 10^−7^ S cm^−1^ at 150 °C).^11,12^ In a later report, a solid solution of Li_2_CO_3_ and Li_3_BO_3_, Li_2.2_C_0.8_B_0.2_O_3_, was described as a superior candidate for assembling the ASSB because of its better conductivity of ≈10^−6^ S cm^−1^ at 25 °C.^13,14^ Two other Li_3_BO_3_-related electrolytes—Li_3_BO_3_–Li_2_SO_4_ amorphous materials^15^ and LISICON-Li_3_BO_3_ amorphous materials^16^—were also attractive candidates, but the ASSBs were assembled according to their deformability due to their amorphous character rather than their thermal stability.

Among Li_3_BO_3_-related families, Lopez-Bermudez *et al.* proposed a solid solution of YBO_3_ and Li_3_BO_3_, that is, Li_6_Y(BO_3_)_3_ (LYBO), as a new crystal-type electrolyte candidate.^17^ The observed ionic conductivity was low at 1.9 × 10^−8^ S cm^−1^ at 50 °C because Li^+^-ion diffusion was permitted only by thermodynamic point-defect formation in the Li_6_Y(BO_3_)_3_. However, based on DFT calculations of the defect-formation energies and Li^+^-ion diffusion barriers, they suggested the structural superiority of Li^+^-ion diffusion in the LYBO-type structure, and mentioned the possibility of conductivity enhancement by aliovalent substitution.^17^

In the present study, we prepared tetravalent ion (Zr^4+^ and Ce^4+^)-doped LYBO-type materials and confirmed an ionic conductivity enhancement to ≈10^−5^ S cm^−1^ at 27 °C. Furthermore, the performance of oxide-based ASSBs was successfully demonstrated with the Zr- and Ce-doped Li_5.875_Y_0.875_Zr_0.1_Ce_0.025_(BO_3_)_3_ (ZC-LYBO) electrolyte because of its thermal stability to layered rock-salt positive electrodes, such as the LiNi_1/3_Mn_1/3_Co_1/3_O_2_ (NMC) positive electrode, and its large electrochemical window.

### Experimental

Tetravalent ion-containing LYBO samples with various molar ratios, Li_6−*x*−*y*_Y_1−*x*−*y*_Zr_*x*_Ce_*y*_(BO_3_)_3_, were prepared using Li_3_BO_3_ (99.9% purity, Toshima Manufacturing Co., Japan), H_3_BO_3_ (99.5% purity, Kanto Chemical Co., Japan), Y_2_O_3_ (99.9% purity, Shin-Etsu Chemical Co., Japan), ZrO_2_ (99.9% purity, Nippon Denko Co., Japan), and CeO_2_ (99.9% purity, Shin-Etsu) as starting materials. An appropriate amount of each compound was weighed to obtain the desired molar ratio, and the mixture was ground using a mechanical milling technique (Pulverisette7, Fritsch). The mixture was pelletized and heated at 650 °C for 12 h. The reacted product was ground and sintered by spark-plasma sintering (SPS) at 600–700 °C for 10 min under a pressure of 30 MPa, to produce the tetravalent ion-containing LYBO compacts.

The structural changes and purities of the samples were evaluated using synchrotron X-ray diffraction (SXRD) analysis (0.5 Å) performed at BL19B2, SPring-8, Sayo, Japan (2019B1881). Rietveld crystal structure refinement of the obtained SXRD patterns was performed using the RIETAN-FP software program,^18^ where a modified split pseudo-Voigt function^19^ was selected for fitting to best represent the profile parameters of the samples. To estimate the Li^+^-ion diffusion pathways in the structure, the refined structural parameters were used as the input file for bond valence site energy (BVSE) calculations, which were performed using SoftBV software.^20,21^ The structural images and maps were drawn using the VESTA software program.^22^

For ion conductivity measurements, both sides of the tetravalent ion-containing LYBO compacts were polished and coated with gold (*via* sputtering) as blocking electrodes. The AC impedance profile was collected with a frequency response analyser (Solartron, 1296) over a frequency range of 0.1 Hz to 1 MHz at 27 °C.

To measure the charge–discharge performance of the ASSB, a composite electrode powder consisting of 50 wt% active material and 50 wt% ZC-LYBO electrolyte was first prepared by mixing in an agate mortar. The active material was either LiNi_1/3_Mn_1/3_Co_1/3_O_2_ (NMC, Toda Kogyo Corp.) or graphite (JFE Chemical Corp.). The composite electrode powder (10 mg) and a Au plate as a current collector were placed on a ZC-LYBO-electrolyte separator of 30 mg in a 10 mm-diameter carbon die, which was heated at 550 °C with an electric current under a pressure of 30 MPa in the SPS process. Lithium foil was used as the counter electrode. A polyethylene oxide (PEO)-based polymer electrolyte film (Osaka Soda, LiTFSA/EO = 0.06) was placed between the lithium foil and the electrolyte side of the composite electrode/electrolyte pellet to reduce the interfacial resistance *via* adhesion.^13^

To confirm the reactivity of the composite electrode after cosintering, laboratory XRD patterns were collected on a diffractometer using Cu Kα radiation (Miniflex600, Rigaku). All XRD analyses were performed in the Bragg–Brentano geometry mode. The microstructure of the composite electrode was observed using field-emission scanning electron microscopy (FE-SEM; JEOL, JSM-5500LV).

To evaluate the electrochemical stability of the ZC-LYBO electrolyte, a metal substrate (Au or Cu) was placed on a ZC-LYBO electrolyte (100 mg) in a 10 mm-diameter carbon die, which was heated at 550 °C with an electric current under a pressure of 30 MPa in the SPS process. Lithium foil and a PEO-based polymer electrolyte film were used as the counter electrode and interfacial connector, respectively.

## Results and discussion

### Structural changes and purities of tetravalent ion-doped LYBO

**Fig. 1:**
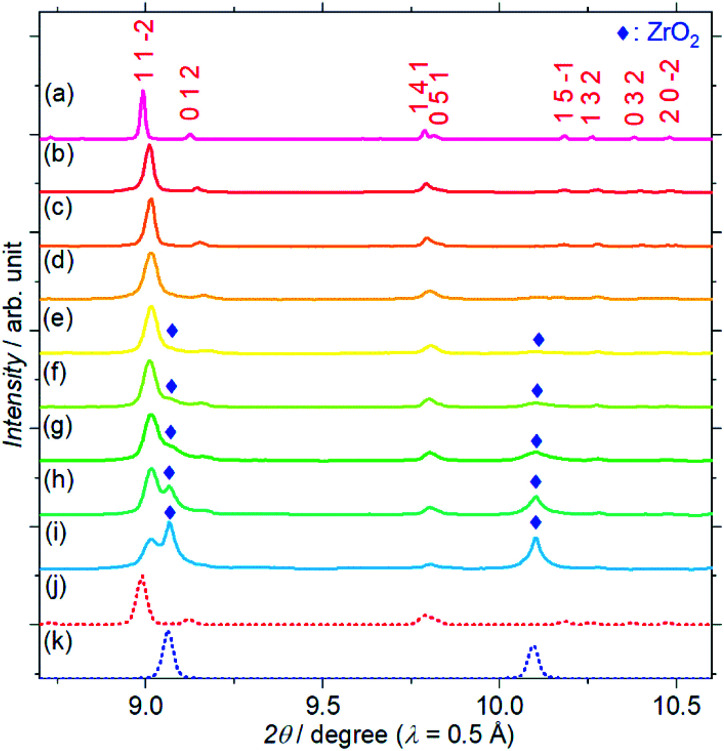
Synchrotron X-ray diffraction patterns of Li_6−*x*_Y_1−*x*_Zr_*x*_(BO_3_)_3_, where *x* = (a) 0, (b) 0.025, (c) 0.05, (d) 0.075, (e) 0.1, (f) 0.2, (g) 0.3, (h) 0.4, (i) 0.6. Simulated patterns for (j) Li_6_Y(BO_3_)_3_ (red dashed line) and (k) ZrO_2_ (blue dashed line) are also shown. The patterns associated with the ZrO_2_ impurity phase are represented by blue diamonds.

Fig. 1 shows the SXRD patterns of the Zr^4+^ ion-containing LYBO samples, Li_6−*x*_Y_1−*x*_Zr_*x*_(BO_3_)_3_ (0 ≤ *x* ≤ 0.6). The *I*(1 1 −2) diffraction peak is clearly shifted to a higher angle with an increasing amount of Zr^4+^ until *x* ≈ 0.1 in Li_6−*x*_Y_1−*x*_Zr_*x*_(BO_3_)_3_ because of the difference in ionic radii: 1.019 Å for Y^3+^ and 0.84 Å for Zr^4+^ in dodecahedral coordination.^23^ In addition, the *I*(1 1 −2) diffraction peak is broadened with an increasing amount of Zr^4+^ ions, which corresponds to the lattice distortion of the LYBO-type structure in addition to the aforementioned mismatch of ionic radii, which will be discussed later. Furthermore, the ZrO_2_ impurity phase begins to appear from *x* ≈ 0.1.

**Fig. 2:**
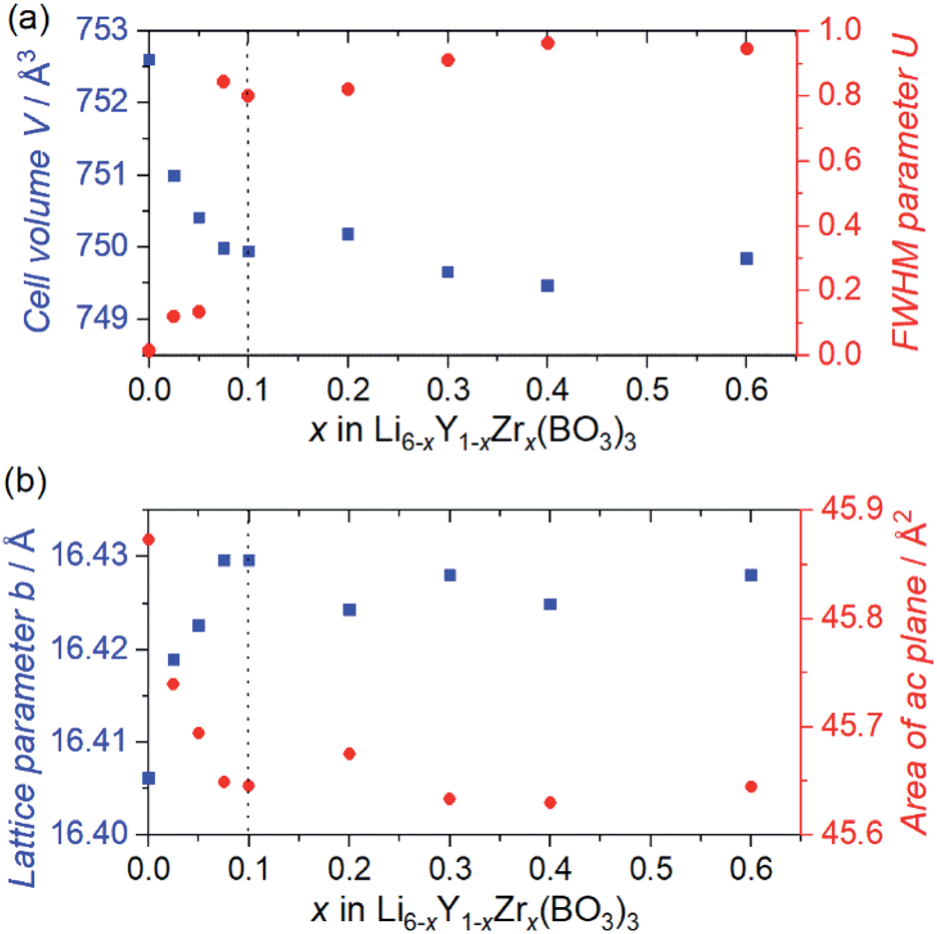
(a) Cell volumes *V* (blue squares) and FWHM parameters *U* (red circles), and (b) lattice parameters *b* (blue squares) and areas of *ac* plane (red circles) for Zr-doped Li_6−*x*_Y_1−*x*_Zr_*x*_(BO_3_)_3_, as estimated by Rietveld refinements of the synchrotron X-ray diffraction patterns.

Rietveld refinements of the SXRD patterns were performed to clarify the structural changes caused by the Zr^4+^ ions. The refined patterns for typical samples and the estimated parameters for all the samples are summarised in Fig. S2(a–c), and Table S1,† respectively. Fig. 2(a) shows the changes in the cell volume and full-width-at-half-maximum (FWHM) parameter *U* for the LYBO-type phases estimated from the refinements. Herein, *U* is one of the simulated components for representing the FWHMs of the total diffraction patterns based on the Caglioti formula^24^ under Rietveld refinement.FWHM^2^ = *U* tan^2^ *θ*_Bragg_ + *V* tan *θ*_Bragg_ + *W*where parameters *V* and *W* mainly depend on the diffractometer characteristics, which were estimated from the refinements of a CeO_2_ reference in advance, and *θ*_Bragg_ is the diffraction peak angle.

**Fig. 3:**
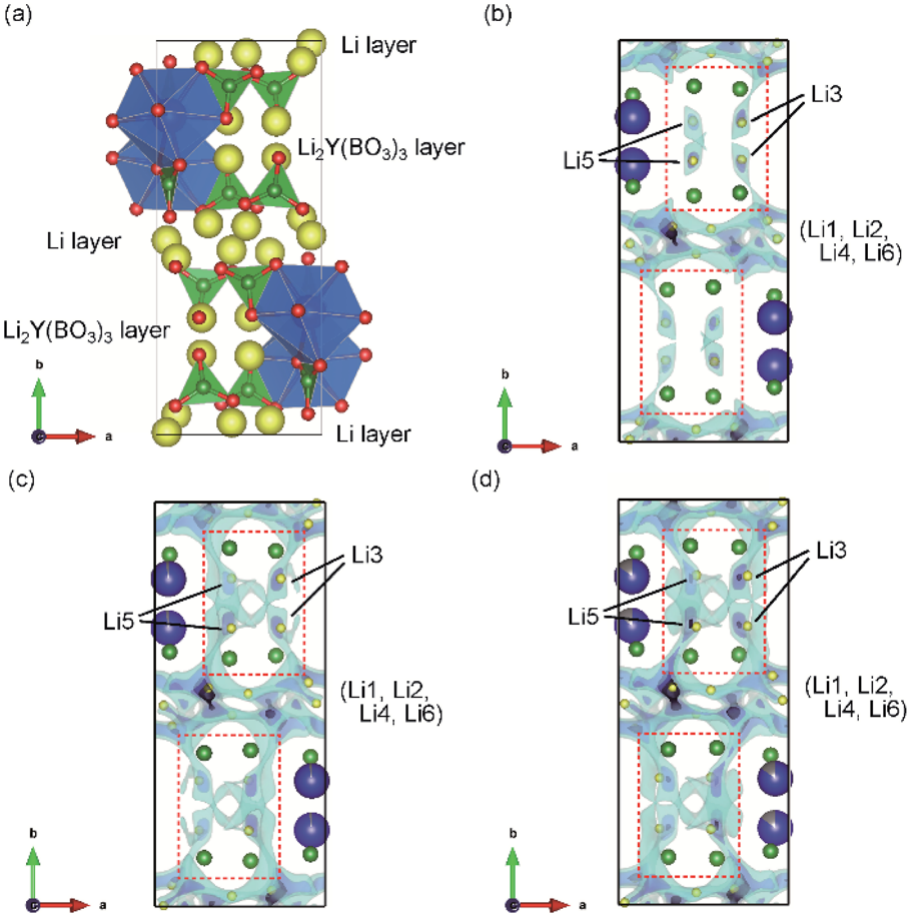
(a) Schematic representation of LYBO-type structure. Blue dodecahedra represent Y(/Zr)O_8_, green triangles represent BO_3_, and yellow balls indicate Li ions. 3D BVSE maps of the *ab* plane for (b) undoped Li_6_Y(BO_3_)_3_, (c) Zr-doped Li_5.975_Y_0.975_Zr_0.025_(BO_3_)_3_, and (d) Zr-doped Li_5.9_Y_0.9_Zr_0.1_(BO_3_)_3_. Translucent clouds of light blue represent the regions of low bond valence site energy for Li^+^ ions.

Thus, *U* represents the FWHM corresponding to the structural distortion in a sample. It should be noted that the structural distortion is difficult to discuss quantitatively in this study because a modified split pseudo-Voigt function^19^ was used to refine the profiles. Despite the difficulties in this case, the definite change in *U* due to the Zr^4+^ ion is strongly related to the structural distortion until *x* ≈ 0.1 in Li_6−*x*_Y_1−*x*_Zr_*x*_(BO_3_)_3_, which is due to the substitution of smaller Zr^4+^ ions at the Y^3+^ sites. The shrinkage of the cell volume until *x* ≈ 0.1 is also proof of Zr^4+^-ion doping. When *x* > 0.1, these changes are suppressed because of the limits of Zr^4+^-ion doping into the LYBO-type structure; thereafter, the ZrO_2_ impurity (and Li–B–O impurities, mainly Li_6_B_4_O_9_) is observed. Moreover, the lattice parameter *b* and area of the *ac* plane are shown separately in Fig. 2(b). The LYBO-type structure has a layered structure that is constructed by the stacking of the Li_2_Y(BO_3_)_3_ layer and the Li layer along the *b*-axis, as shown in Fig. 3(a). The shrinkage of the YO_8_ dodecahedra upon doping with smaller Zr^4+^ ions until *x* ≈ 0.1 directly reduces the area of the *ac* plane. On the other hand, the lattice parameter *b* is expanded until *x* ≈ 0.1 because of the decrease in Coulomb repulsion force at the Li layer due to the lack of Li^+^ ions under the doping of Zr^4+^ ions into Li_6−*x*_Y_1−*x*_Zr_*x*_(BO_3_)_3_. This tendency is similar to the change in the lattice parameter *c* during the electrochemical de-lithiation of layered rock-salt Li_1−*x*_CoO_2_.^25^ Therefore, Li^+^ ions are likely to be removed from the Li layer rather than from the Li_2_Y(/Zr)(BO_3_)_3_ layer by doping Zr^4+^ ions into Li_6−*x*_Y_1−*x*_Zr_*x*_(BO_3_)_3_, which is supported by previous DFT calculation results in which the Li defect energies at the Li layer are lower than those at the Li_2_Y(BO_3_)_3_ layer in Li_6_Y(BO_3_)_3_.^17^

### Li-ion conductive pathways

The Li^+^-ion diffusion pathways in the LYBO-type structure were examined from bond-valence site energy (BVSE) maps; the *ab* and *ac* planes are shown in Fig. 3(b–d) and S3(b–d),† respectively. The BVSE maps present the theoretical mobile Li^+^-ion pathways as clouds with low bond valence site energies. Although the mobile Li^+^ ions form a 2D diffusion pathway network at the Li layer on the *ac* plane [Fig. S3(b)†], it is difficult for Li^+^ ions to diffuse along the *b* axis *via* Li3 or Li5 sites in undoped Li_6_Y(BO_3_)_3_, as shown by the red dashed boxes in Fig. 3(b). This was previously suggested from DFT calculations.^17^ As shown in Fig. 3(c, d), the expansion of the diffusion pathways along the *b*-axis could be induced by doping Zr^4+^ ions into Li_6−*x*_Y_1−*x*_Zr_*x*_(BO_3_)_3_, which indicates that the structural distortion in the Li_2_Y(/Zr)(BO_3_)_3_ layer contributed to expanding the diffusion pathways. The 2D diffusion pathways in the Li layer also become thin, as can be confirmed from Fig. S3(c, d).† These results indicate that Li^+^ ions are more easily diffused by the structural distortion associated with doping Zr^4+^ ions into Li_6−*x*_Y_1−*x*_Zr_*x*_(BO_3_)_3_. The activation energies for the 2D and 3D pathways estimated from BVSE decrease from 0.57 and 0.69 eV to 0.49 and 0.59 eV, respectively, even by doping with only *x* = 0.025 Zr^4+^ ions into Li_6−*x*_Y_1−*x*_Zr_*x*_(BO_3_)_3_.

### Ionic conductivities

**Fig. 4:**
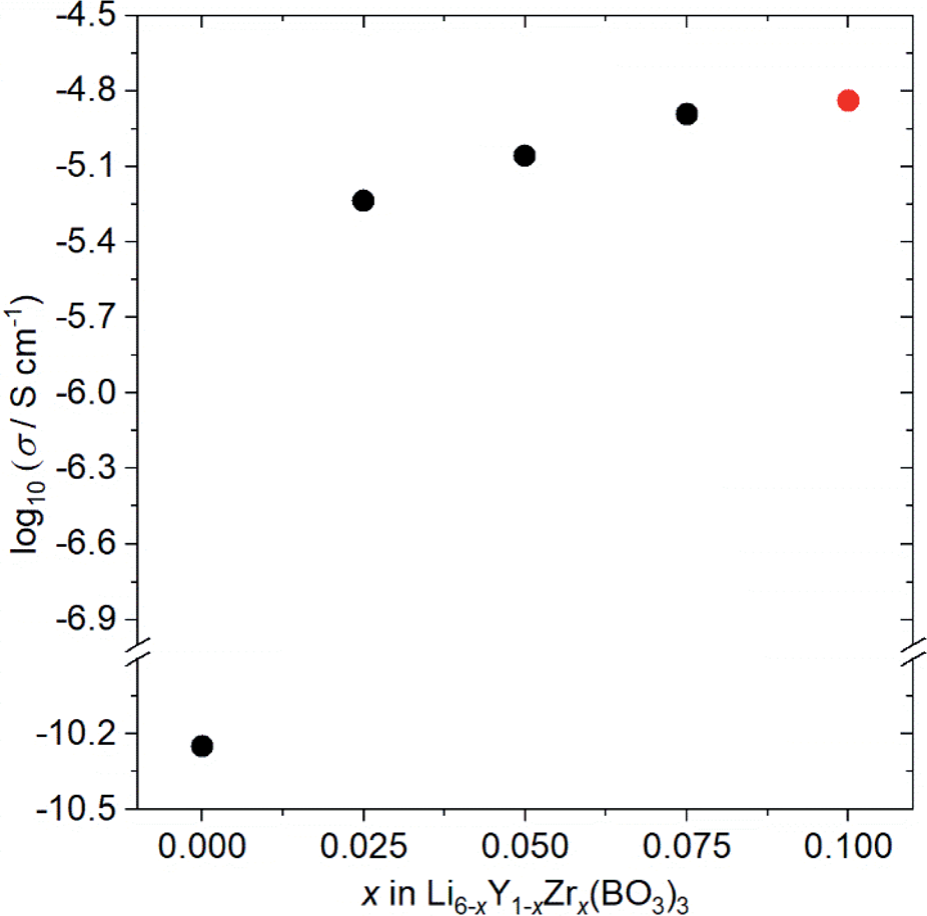
Ionic conductivity dependence on the composition of Li_6−*x*_Y_1−*x*_Zr_*x*_(BO_3_)_3_ (0 ≤ *x* ≤ 0.10) at 27 °C. Li_5.9_Y_0.9_Zr_0.1_(BO_3_)_3_ (*x* = 0.10) contains small amount of ZrO_2_ impurity (red circle).

Fig. 4 shows the ionic conductivity dependence when Zr^4+^ ions are incorporated into Li_6−*x*_Y_1−*x*_Zr_*x*_(BO_3_)_3_ (0 ≤ *x* ≤ 0.1). The observed conductivity at 27 °C is drastically enhanced from 5.6 × 10^−11^ to 5.8 × 10^−6^ S cm^−1^ by doping with *x* = 0.025 Zr^4+^ ions into Li_6−*x*_Y_1−*x*_Zr_*x*_(BO_3_)_3_. Li^+^-ion diffusion in Li-ion fully occupied Li_6_Y(BO_3_)_3_ occurs only through the formation of a few thermodynamic point defects. In contrast, the substitution of tetravalent Zr^4+^ ions in the Y^3+^ sites results in the intentional formation of vacancies at Li sites in the structure, which contributes to the conductivity enhancement. Additionally, the observed conductivities are sensitive to the relative densities of the Li_6−*x*_Y_1−*x*_Zr_*x*_(BO_3_)_3_ compacts, which are summarised in Table S2.† For example, the observed conductivity in a low-density Li_3.5_Ge_0.75_S_0.25_O_4_ compact (79%) was over a hundred times lower than a high density compact (∼90%).^26^ Therefore, the increase in relative density upon doping with Zr^4+^ ions, from 77% (*x* = 0) to ∼90% (*x* > 0), also contributes to the ionic conductivity enhancement. Moreover, the expansion of 3D diffusion pathways by Zr^4+^ doping, as estimated by the Rietveld analyses, would also affect the enhancement. To confirm this effect, the structure and conductivity of Ce-doped Li_5.975_Y_0.975_Ce_0.025_(BO_3_)_3_ were also examined. The ionic radius for the Ce^4+^ ion dopant in dodecahedral coordination is 0.97 Å, which is more comparable to that for the Y^3+^ ion (1.019 Å) than the Zr^4+^ ion dopant (0.84 Å).^23^ Therefore, the lattice distortion by doping the tetravalent ion is supressed; the lattice distortion in Ce-doped Li_5.975_Y_0.975_Ce_0.025_(BO_3_)_3_ (*U* = 0.02354 deg^2^) is closer to that in undoped Li_6_Y(BO_3_)_3_ (*U* = 0.01488 deg^2^) than that in Zr-doped Li_5.975_Y_0.975_Zr_0.025_(BO_3_)_3_ (*U* = 0.119 deg^2^), as can be confirmed in Table S1.† Considering the conductivities shown in Table S3,† that of the LYBO-type structure is drastically enhanced by 0.025 Ce^4+^ doping (Li_5.975_Y_0.975_Ce_0.025_(BO_3_)_3_; *σ* = 6.9 × 10^−7^ S cm^−1^ at 27 °C), which is due to the formation of vacancies at Li sites in the structure and the increase in compact densification, as discussed above. However, the conductivity of the Ce^4+^-ion doped sample is over ten times lower than that of the Zr^4+^-ion doped one. This difference indicates that the structural distortion associated with the doping of the larger Zr^4+^ ions certainly affects the enhancement of the conductivity. The differences in the Li^+^-ion diffusion pathways estimated from the BVSE maps also support the aforementioned results: for the less-structurally-distorted Ce^4+^-ion doped sample (Fig. S4(a, b)†), the diffusion pathway along the *b*-axis in the BVSE map is nearly unchanged. Therefore, Zr^4+^-ion doping exerts three types of effects that enhance the conductivity of the LYBO-type structure: (1) the formation of vacancies at Li sites, (2) an increase in compact densification, and (3) an increase in the Li^+^-ion conduction pathways in the LYBO phase associated with structural distortion. The highest conductivity in Zr^4+^-ion-doped Li_6−*x*_Y_1−*x*_Zr_*x*_(BO_3_)_3_ at 27 °C is 1.4 × 10^−5^ S cm^−1^ at *x* = 0.10. However, Zr^4+^-ion doping achieves a limit at *x* ≈ 0.1 in Li_6−*x*_Y_1−*x*_Zr_*x*_(BO_3_)_3_, which would be due to the large mismatch of ionic radii. Therefore, the conductivity decreases for *x* > 0.1 (Table S3†), with increases in the amounts of ZrO_2_ and Li–B–O impurities.

The conductivity of Zr,Ce-doped Li_5.875_Y_0.875_Zr_0.1_Ce_0.025_Zr_0.1_(BO_3_)_3_ (ZC-LYBO) was also measured, and the obtained value of 1.7 × 10^−5^ S cm^−1^ at 27 °C is the highest conductivity of LYBO-type oxides to date. The structural information estimated from Rietveld analysis, Li^+^-ion diffusion pathways estimated from BVSE maps, and relative densities of the compacts are summarised in Table S1, Fig. S3(d), and Table S2.† Compared with Zr-doped Li_5.9_Y_0.9_Zr_0.1_(BO_3_)_3_, a slight enhancement in conductivity is confirmed, but the reason is difficult to determine in the present study. The conductivity measurement results indicate the possibility of further enhancement by other aliovalent substitutions. Moreover, the control of structural distortion is one of the keys for enhancing the conductivity of the LYBO-type structure. It should be noted that the activation energies estimated from Arrhenius plots (Fig. S5†) were 0.43 eV for both Zr-doped Li_5.9_Y_0.9_Zr_0.1_(BO_3_)_3_ and Zr,Ce-doped Li_5.875_Y_0.875_Ce_0.025_Zr_0.1_(BO_3_)_3_ (ZC-LYBO). Based on its high conductivity, ZC-LYBO was used as the electrolyte in subsequent ASSB studies.

### Thermal stability after cosintering with layered rock-salt oxide active material

**Fig. 5:**
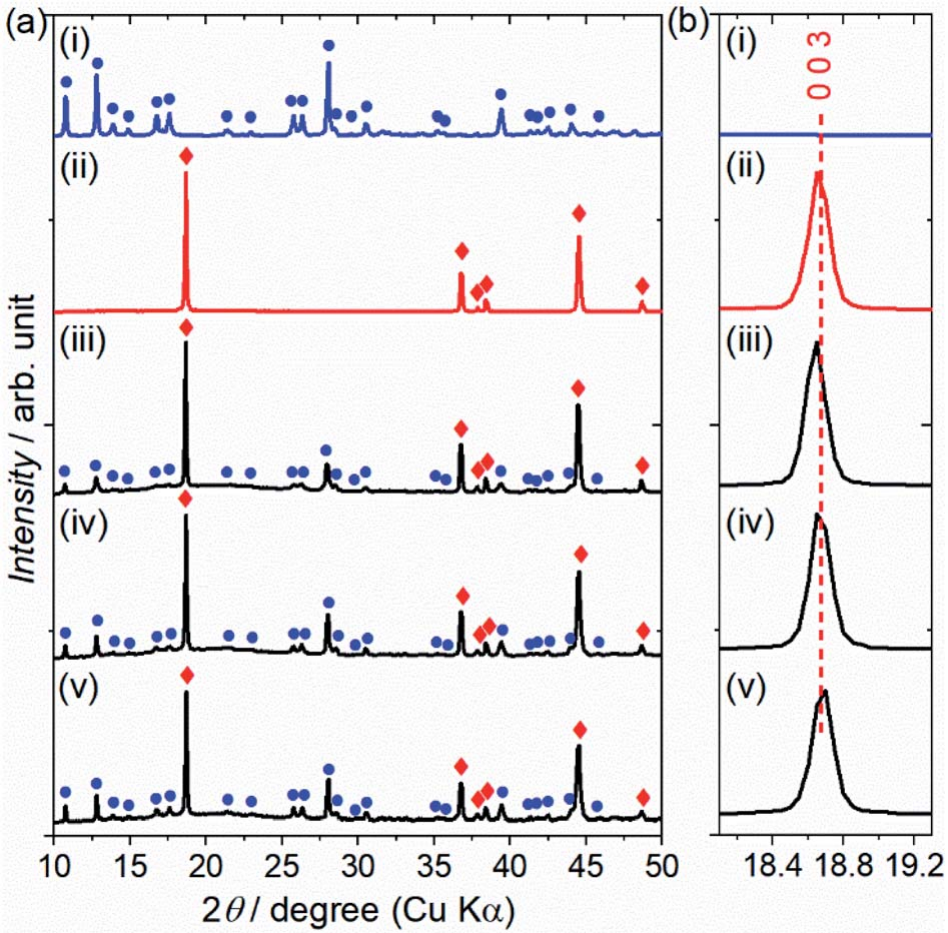
(a) Laboratory XRD patterns of (i) ZC-LYBO, (ii) NMC, and the NMC–ZC-LYBO combinations after SPS at (iii) 450 °C, (iv) 550 °C, and (v) 650 °C. Typical diffraction peaks for ZC-LYBO and NMC are represented by blue circles and red diamonds, respectively. (b) The significant diffraction positions at *I*(0 0 3) for NMC.

The reactivity of the layered rock-salt LiNi_1/3_Mn_1/3_Co_1/3_O_2_ (NMC) electrode and ZC-LYBO electrolyte after cosintering was evaluated by laboratory XRD patterns (Fig. 5(a)). None of the XRD peaks are associated with impurities, and no significant peak shifts after co-sintering are observed for either the NMC electrode or ZC-LYBO electrolyte, as shown for a typical peak in Fig. 5(b). Thus, interdiffusion between the NMC and ZC-LYBO was minimal, with no penetration of the bulk materials during cosintering. This result indicates that ZC-LYBO is thermally stable with a layered rock-salt NMC electrode.

**Fig. 6:**
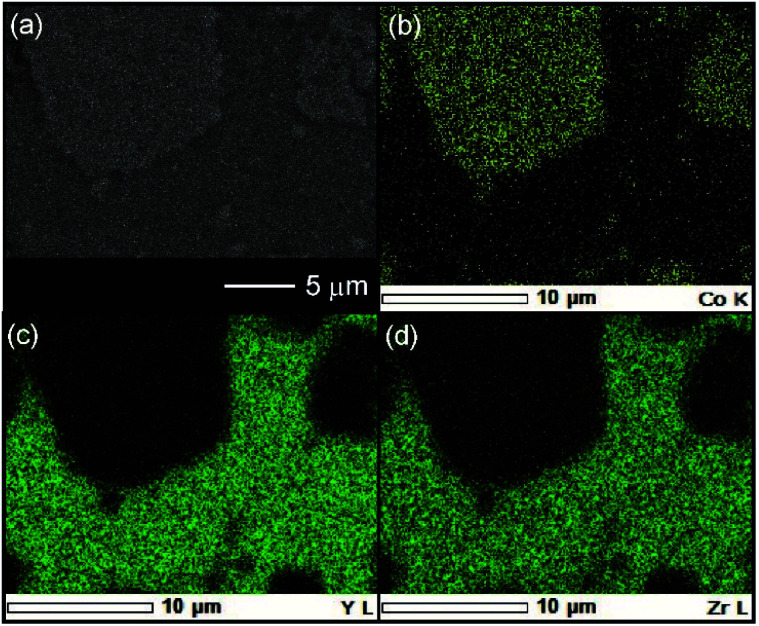
(a) Cross-sectional SEM image at the NMC–ZC-LYBO interface after SPS at 550 °C, with the corresponding EDX elemental maps for (b) Co, (c) Y, and (d) Zr.

The cross-sectional microstructure of the NMC + ZC-LYBO composite electrode after the 550 °C SPS process was also investigated. As shown in Fig. 6(a), the NMC electrode particles (light grey) are embedded in the ZC-LYBO electrolyte (dark grey). Moreover, no micropores are present near the NMC electrodes because the ZC-LYBO electrolyte was densified after SPS. No evidence of diffusion is found in the EDX mappings for Co [NMC electrode, Fig. 6(b)], Y, and Zr [ZC-LYBO electrolyte, Fig. 6(c and d)]. This indicates that the ZC-LYBO electrolyte and NMC electrode can be co-sintered without the formation of any interfacial impurities, which is one of the rare characteristics of the electrolyte and is not possible with other popular oxide-electrolyte candidates (*e.g.* perovskite-type conductors, NASICONs, and garnet-type conductors).

**Fig. 7:**
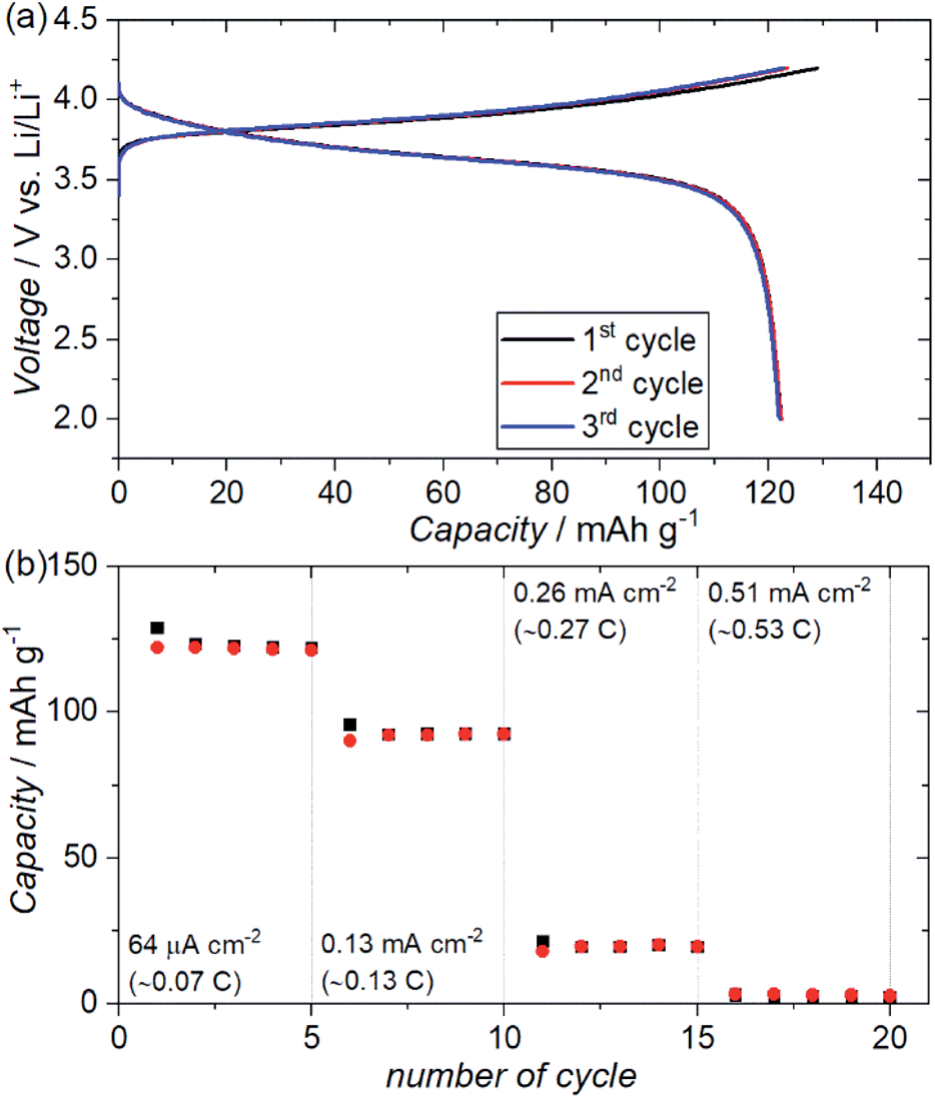
(a) Electrochemical charge–discharge profiles of an ASSB (Au|NMC + ZC-LYBO composite electrode|ZC-LYBO separator|dry polymer|Li metal) prepared *via* SPS at 550 °C over three charge–discharge cycles at a constant current of 64 μA cm^−2^ at 60 °C. (b) Charge–discharge capacity retentions of the ASSB at different current densities at 60 °C.

Fig. 7(a) shows the charge–discharge profiles for an ASSB (NMC + ZC-LYBO composite electrode|ZC-LYBO separator|dry polymer|Li metal) assembled *via* SPS. A reversible capacity of ∼120 mA h g^−1^ is observed at 60 °C owing to the impurity-free sintered interface between the NMC and ZC-LYBO. The charge–discharge capacity retentions at various current densities are shown in Fig. 7(b). Although the capacity decreases at higher current densities, relatively stable capacity retention under cycling for each current density is observed for the ASSB. In fact, the rate performance of the ASSB using the ZC-LYBO electrolyte is lower than that using LISICON-type Li_3.5_Ge_0.5_V_0.5_O_4_ (LGVO), which has also been reported as a thermally stable electrolyte with an NMC electrode.^27^ This is due to the lower conductivity of ZC-LYBO compared to Li_3.5_Ge_0.5_V_0.5_O_4_ (*i.e.* 9.6 × 10^−5^ S cm^−1^ at 25 °C).^27^ However, a broader acceptance of aliovalent substitutions in the less-reported LYBO-type electrolytes could lead to the development of a more highly ion-conductive LYBO family, analogous to the advances achieved with other electrolyte candidates.^1^

### Electrochemical window

**Fig. 8:**
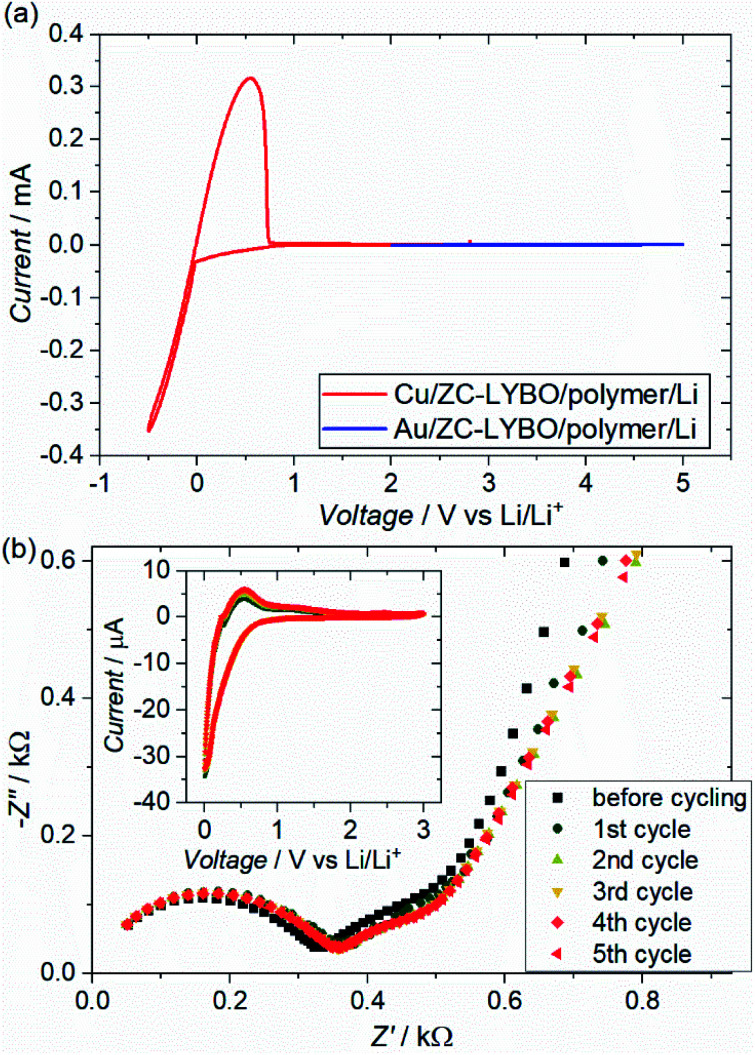
(a) Cyclic voltammograms (CVs) of test cells (metal substrate|ZC-LYBO electrolyte|dry polymer|Li metal) prepared by the 550 °C-SPS process at a scan rate of 100 μV s^−1^ at 60 °C. Cu and Au metal substrates were used for testing the cathodic and the anodic reactions, respectively. (b) Nyquist plots of the test cell using Cu substrate at 60 °C after the CV cycling and the CV profile at each cycle.

Fig. 8(a) shows the cyclic voltammograms (CVs) of test cells (metal substrate|ZC-LYBO electrolyte|dry polymer|Li metal) assembled by SPS. We evaluated the electrochemical stability of the ZC-LYBO electrolyte in the low-voltage range from 3.0 to −0.5 V *vs.* Li/Li^+^ and in the high-voltage range from 2.0 to 5.0 V *vs.* Li/Li^+^ with a Cu substrate (red) and an Au substrate (blue), respectively. The ZC-LYBO electrolyte is stable at high voltages. Au is kinetically stable within a wide electrochemical window but produces Li_*x*_Au_*y*_ alloys at voltages below 0.2 V *vs.* Li/Li^+^ when in contact with Li metal.^28,29^ Therefore, Cu was used as the substrate to evaluate the low-voltage stability.^30^ Cathodic and anodic currents corresponding to Li metal deposition (Li^+^ + e^−^ → Li) and dissolution (Li → Li^+^ + e^−^), respectively, are observed at 0 V *vs.* Li/Li^+^. Although the ZC-LYBO electrolyte is nearly electrochemically stable in the range from 0.0 to 5.0 V *vs.* Li/Li^+^, a slight cathodic peak can be observed below ≈1.0 V *vs.* Li/Li^+^. Thus, the CVs under 5 cycles in the range from 3.0 to 0.0 V *vs.* Li/Li^+^ were also recorded (Fig. 8(b), insert). A slight cathodic peak continuously appears in each CV cycle and is irreversible, which would result in decomposition on the surface of the ZC-LYBO electrolyte at low voltage. Fig. 8(b) shows the conductivity change for the LYBO electrolyte after each CV cycle. Despite the continuous appearance of a cathodic peak, the Nyquist plots, including the resistance in the ZC-LYBO electrolyte, change less. Therefore, ZC-LYBO can also be used as an electrolyte with a low-voltage negative electrode.

**Fig. 9:**
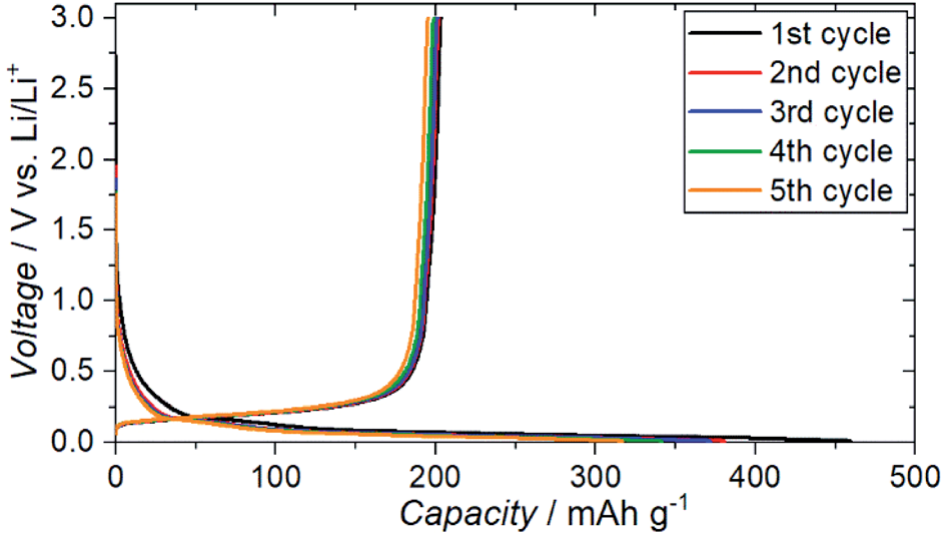
Electrochemical charge–discharge profiles of Cu|graphite + ZC-LYBO composite electrode|ZC-LYBO separator|dry polymer|Li metal.

The charge–discharge profiles for a battery (Cu|graphite negative electrode + ZC-LYBO composite electrode|ZC-LYBO separator|dry polymer|Li metal) assembled by the SPS process are shown in Fig. 9. A typical reversible Li-ion (de)intercalation reaction for a graphite negative electrode is observed, and a first discharge capacity of ∼200 mA h g^−1^ was obtained along with a large irreversible capacity for each cycle. The irreversible capacity is related to the slight anodic reaction below 1.0 V Li/Li^+^ (Fig. 8).

Among the various oxide-type electrolytes, ZC-LYBO is rare; it is possible to assemble ASSBs by sintering with both layered lock-salt positive electrodes such as NMC and low-voltage negative electrodes such as graphite. LISICON-type Li_3.5_Ge_0.5_V_0.5_O_4_ (LGVO) can also be cosintered with layered rock-salt positive electrodes but decompose at low voltage because of the presence of Ge^4+^ and V^5+^ ions.^27^ In fact, other Li_3_BO_3_-related families such as Li_2.2_C_0.8_B_0.2_O_3_ (ref. 13 and 14) and LISICON-Li_3_BO_3_ (ref. 16) are stable at low voltage, although conductivities are limited to ≈10^−6^ S cm^−1^ at 25 °C. Therefore, the LYBO-type crystal system may be an attractive candidate for assembling ASSBs by sintering after further enhancing the conductivity *via* aliovalent substitution. Even the ZC-LYBO in the present study can be used as an ion-conductive ceramic binder for sintering ASSBs instead of low-ion-conductive Li_3_BO_3_,^12^ which has been used to connect LiCoO_2_ electrodes and high-ion-conductive garnet-type electrolytes.^11^

## Conclusions

We substituted tetravalent ions (Zr^4+^ and Ce^4+^) for the Y^3+^ sites in the Li_6_Y(BO_3_)_3_ (LYBO)-type structure to enhance conductivity. The ionic conductivities of Zr-doped Li_5.9_Y_0.9_Zr_0.1_(BO_3_)_3_ and Zr,Ce-doped Li_5.875_Y_0.875_Ce_0.025_Zr_0.1_(BO_3_)_3_ (ZC-LYBO) were respectively 1.4 × 10^−5^ and 1.7 × 10^−5^ S cm^−1^ at 27 °C, which are some of highest conductivities reported for Li_3_BO_3_-related electrolyte candidates. The effects of Zr^4+^-ion doping on the conductivity of the LYBO-type structure were revealed from the structural information estimated from Rietveld analysis, relative density of compacts, and Li^+^-ion diffusion pathways estimated from BVSE maps, and included: (1) the formation of vacancies at Li sites, (2) the increase of compact densification, and (3) an increase in the Li^+^-ion conduction pathways in the LYBO phase associated with structural distortion.

Furthermore, sintered ASSBs using ZC-LYBO as an electrolyte successfully performed with both a LiNi_1/3_Mn_1/3_Co_1/3_O_2_ (NMC) positive electrode and a graphite negative electrode. This was due to the thermal stability of the layered rock-salt oxide and the electrochemical stability of the ZC-LYBO at low voltages. The conductivity of the LYBO-type electrolyte could be further enhanced by other aliovalent substitutions for the practical use of sintered ASSBs. The control of the structural distortion associated with the dopant size, which was revealed in this study, could play an important role in this enhancement.

## Conflicts of interest

There are no conflicts to declare.

## Supplementary Material

RA-011-D1RA02191E-s001
