# Supplementary material for: Zr- and Ce-doped Li6Y(BO3)3 electrolyte for all-solid-state lithium-ion battery
Source: RSC Adv. 2021 May 5;11(27):16530–6. doi: 10.1039/d1ra02191e (PMC9031198; doi:10.1039/d1ra02191e)
Supplement: RA-011-D1RA02191E-s001 [file RA-011-D1RA02191E-s001.pdf]

Electronic Supplementary Material (ESI) for RSC Advances.
This journal is © The Royal Society of Chemistry 2021

Supporting Information

Zr- and Ce-Doped $Li_6Y(BO_3)_3$ Electrolyte for All-Solid-State Lithium-Ion Battery

Toyoki Okumura,[a,*] Yoshitaka Shiba,[b] Noriko Sakamoto,[b] Takeshi Kobayashi,[b] Saori Hashimoto,[b] Kentaro Doguchi,[b] Harunobu Ogaki,[b] Tomonari Takeuchi,[a] Hironori Kobayashi[a]

a. *Research Institute of Electrochemical Energy, National Institute of Advanced Industrial Science and Technology (AIST), Midorigaoka 1-8-31, Ikeda, Osaka 563-8577, Japan E-mail: toyoki-okumura@aist.go.jp*

b. *Development Division, Canon Optron Inc., 1744-1 Kanakubo, Yuki, Ibaraki 307-0015, Japan*

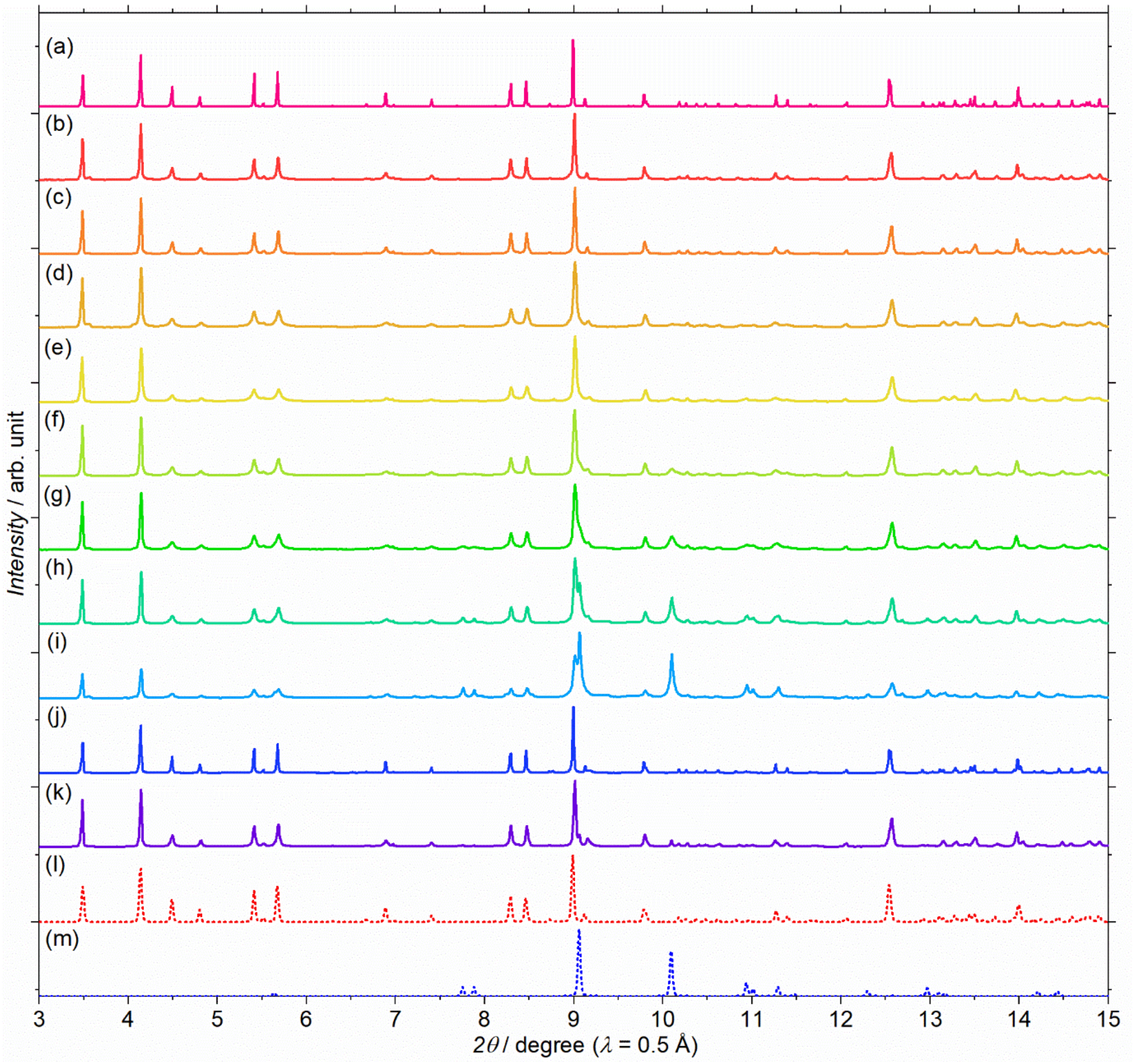

Fig. S1 Expanded synchrotron X-ray diffraction patterns of $Li_{6\text{-}x}Y_{1\text{-}x}Zr_x(BO_3)_3$, where x = (a) 0, (b) 0.025, (c) 0.05, (d) 0.075, (e) 0.1, (f) 0.2, (g) 0.3, (h) 0.4, (i) 0.6, and those of $Li_{5.975\text{-}x}Y_{0.975\text{-}x}Zr_xCe_{0.025}(BO_3)_3$, x = (j) 0, (k) 0.1. Simulated patterns for (l) $Li_6Y(BO_3)_3$ (red dashed line) and (m) ZrO_2 (blue dashed line) are also shown.

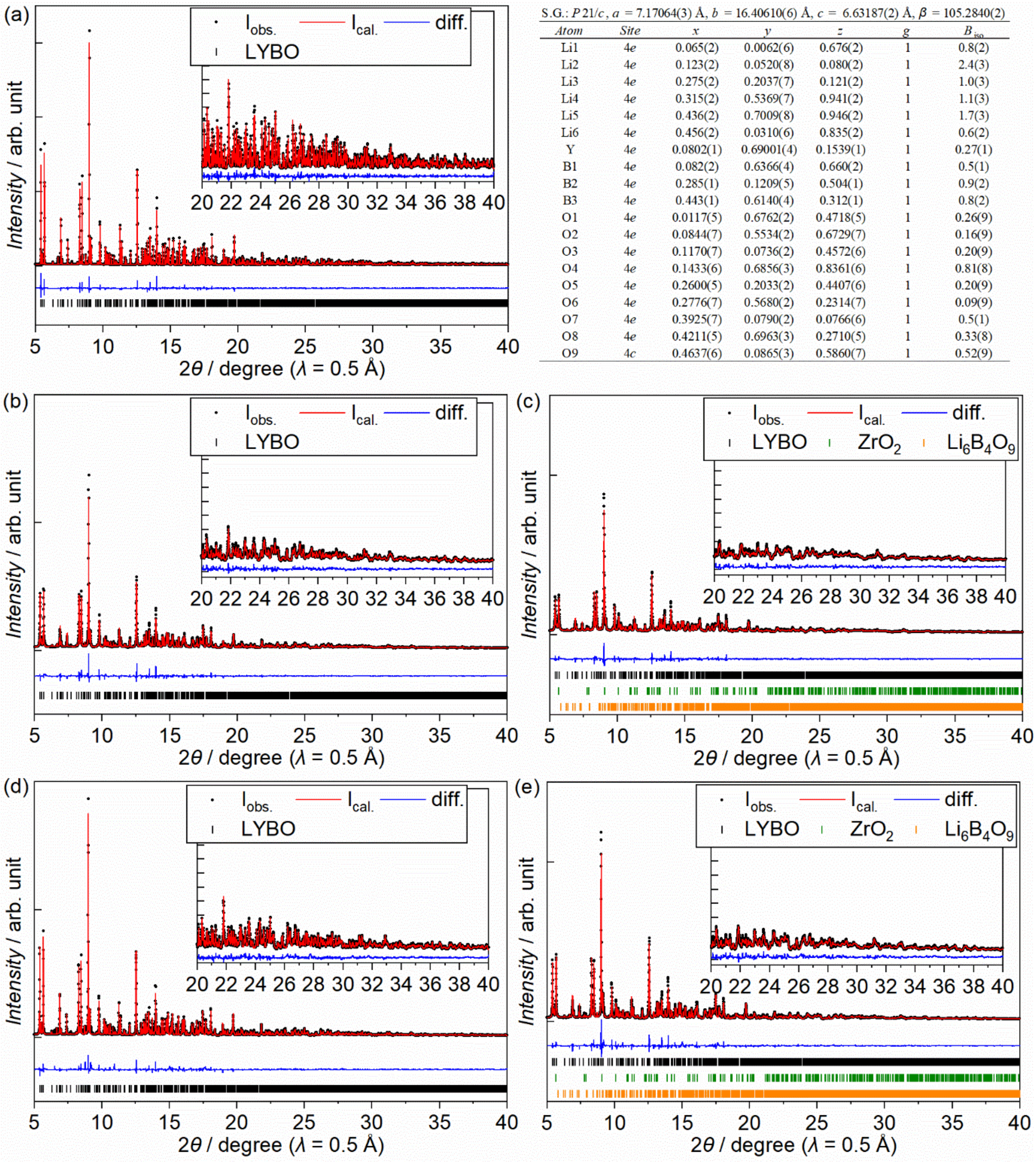

S.G.: $P2_1/c$, a = 7.17064(3) Å, b = 16.40610(6) Å, c = 6.63187(2) Å, β = 105.2840(2)

Atom	*Site*	*x*	*y*	*z*	*g*	B_{iso}
Li1	4*e*	0.065(2)	0.0062(6)	0.676(2)	1	0.8(2)
Li2	4*e*	0.123(2)	0.0520(8)	0.080(2)	1	2.4(3)
Li3	4*e*	0.275(2)	0.2037(7)	0.121(2)	1	1.0(3)
Li4	4*e*	0.315(2)	0.5369(7)	0.941(2)	1	1.1(3)
Li5	4*e*	0.436(2)	0.7009(8)	0.946(2)	1	1.7(3)
Li6	4*e*	0.456(2)	0.0310(6)	0.835(2)	1	0.6(2)
Y	4*e*	0.0802(1)	0.69001(4)	0.1539(1)	1	0.27(1)
B1	4*e*	0.082(2)	0.6366(4)	0.660(2)	1	0.5(1)
B2	4*e*	0.285(1)	0.1209(5)	0.504(1)	1	0.9(2)
B3	4*e*	0.443(1)	0.6140(4)	0.312(1)	1	0.8(2)
O1	4*e*	0.0117(5)	0.6762(2)	0.4718(5)	1	0.26(9)
O2	4*e*	0.0844(7)	0.5534(2)	0.6729(7)	1	0.16(9)
O3	4*e*	0.1170(7)	0.0736(2)	0.4572(6)	1	0.20(9)
O4	4*e*	0.1433(6)	0.6856(3)	0.8361(6)	1	0.81(8)
O5	4*e*	0.2600(5)	0.2033(2)	0.4407(6)	1	0.20(9)
O6	4*e*	0.2776(7)	0.5680(2)	0.2314(7)	1	0.09(9)
O7	4*e*	0.3925(7)	0.0790(2)	0.0766(6)	1	0.5(1)
O8	4*e*	0.4211(5)	0.6963(3)	0.2710(5)	1	0.33(8)
O9	4*c*	0.4637(6)	0.0865(3)	0.5860(7)	1	0.52(9)

Fig. S2 Rietveld refinements of synchrotron X-ray diffraction patterns for (a) undoped $Li_6Y(BO_3)_3$, (b) Zr-doped $Li_{5.975}Y_{0.975}Zr_{0.025}(BO_3)_3$, (c) Zr-doped $Li_{5.9}Y_{0.9}Zr_{0.1}(BO_3)_3$, (d) Ce-doped $Li_{5.975}Y_{0.975}Ce_{0.025}(BO_3)_3$, and (e) Zr,Ce-doped $Li_{5.875}Y_{0.875}Zr_{0.1}Ce_{0.025}$ $(BO_3)_3$ (ZC-LYBO). The observed, calculated, and difference plots are shown by black circles, red solid lines, and blue solid lines, respectively. Diffraction positions are indicated by colored bars: black (LYBO-type phase), green (ZrO_2 impurity), and orange ($Li_6B_4O_9$ impurity).

Table S1 Lattice parameters (*a*, *b*, *c*, *β*), FWHM parameters *U* and reliability factors (R_{wp}, *S*) estimated under the Rietveld refinements for Zr-doped $Li_{6-x}Y_{1-x}Zr_x(BO_3)_3$, Ce-doped $Li_{5.975}Y_{0.975}Ce_{0.025}(BO_3)_3$, and Zr,Ce-doped $Li_{5.875}Y_{0.875}Zr_{0.1}Ce_{0.025}(BO_3)_3$ (ZC-LYBO)

$Li_{6-x}Y_{1-x}Zr_x(BO_3)_3$	*a* / Å	*b* / Å	*c* / Å	*β* / deg.	*U* / deg.^{2}	R_{wp} / %	*S* / -
x = 0	7.17064(3)	16.40610(6)	6.63187(2)	105.2840(2)	0.014880(6)	6.216	3.9692
x = 0.025	7.1665(2)	16.4189(3)	6.6174(1)	105.315(1)	0.1190(1)	7.556	4.7433
x = 0.05	7.1651(2)	16.4226(3)	6.6133(1)	105.351(1)	0.1351(1)	8.344	5.1733
x = 0.075	7.1676(3)	16.4297(5)	6.6087(3)	105.489(3)	0.8430(4)	6.951	4.3511
x = 0.1*)	7.1667(2)	16.4293(5)	6.6083(2)	105.409(2)	0.7902(3)	7.005	4.1655
x = 0.2*)	7.1662(3)	16.4243(5)	6.6128(2)	105.452(2)	0.8212(4)	7.666	4.5861
x = 0.3*)	7.1667(3)	16.4281(6)	6.6077(3)	105.499(3)	0.9101(5)	7.379	4.6650
x = 0.4*)	7.1651(3)	16.4249(6)	6.6081(3)	105.481(3)	0.9636(5)	7.732	4.8452
x = 0.6*)	7.1658(6)	16.428(1)	6.6101(5)	105.500(6)	0.945(1)	8.398	5.6458
$Li_{6-y}Y_{1-y}Ce_y(BO_3)_3$	*a* / Å	*b* / Å	*c* / Å	*β* / deg.	*U* / deg.^{2}	R_{wp} / %	*S* / -
y = 0.025	7.17069(9)	16.4143(1)	6.62903(8)	105.2929(8)	0.023540(3)	9.644	5.8044
$Li_{6-x-y}Y_{1-x-y}Zr_xCe_y(BO_3)_3$	*a* / Å	*b* / Å	*c* / Å	*β* / deg.	*U* / deg.^{2}	R_{wp} / %	*S* / -
x = 0.1, *y* = 0.025*)	7.1650(2)	16.4250(4)	6.6120(2)	105.397(2)	0.3081(2)	8.440	5.1604

*) Diffraction patterns for ZrO_2 impurity and $Li_6B_2O_9$ impurity were also refined for representing profiles.

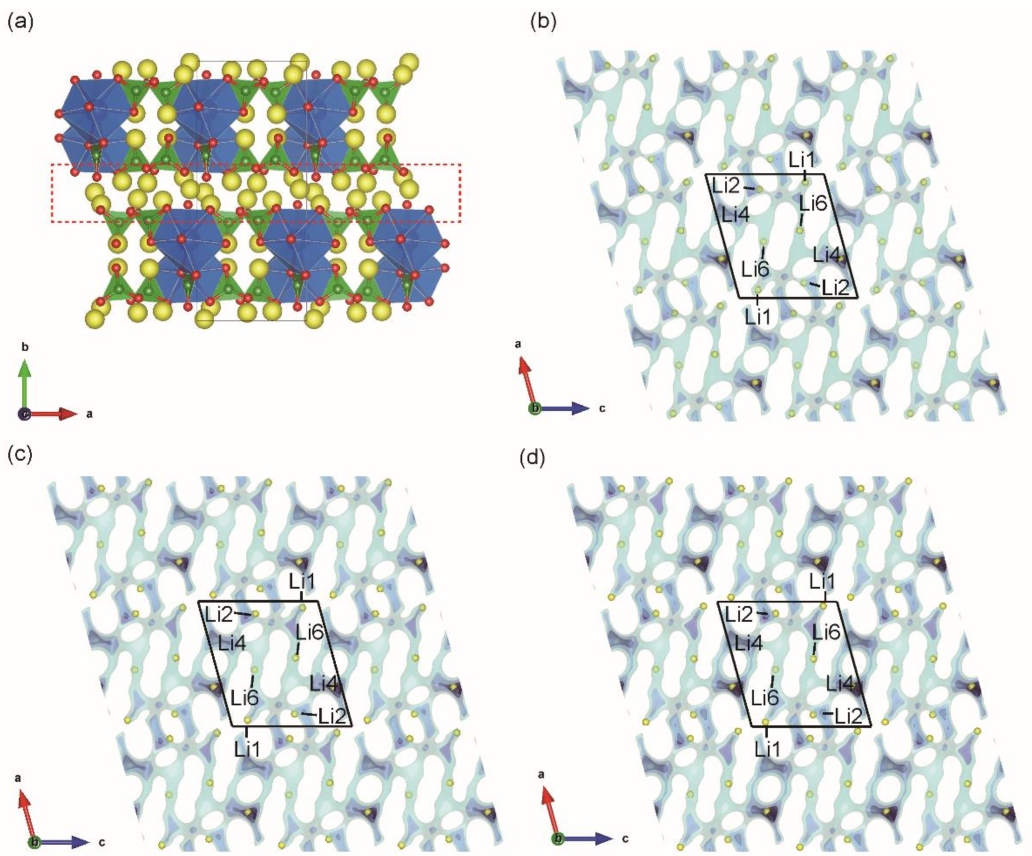

Fig. S3 (a) Schematic representation of LYBO-type structure. Blue dodecahedra represent $Y(/Zr)O_8$, green triangles represent BO_3, and yellow balls indicate Li ions. Focused Li layer along the *ac* plane is enclosed by a red dashed square. 3D BVSE maps of the *ac* plane for (b) undoped $Li_6Y(BO_3)_3$, (c) Zr-doped $Li_{5.975}Y_{0.975}Zr_{0.025}(BO_3)_3$, and (d) Zr-doped $Li_{5.9}Y_{0.9}Zr_{0.1}(BO_3)_3$.

Table S2 Relative densities after sintering for Zr-doped $Li_{6-x}Y_{1-x}Zr_x(BO_3)_3$, Ce-doped $Li_{5.975}Y_{0.975}Ce_{0.025}(BO_3)_3$, and Zr,Ce-doped $Li_{5.875}Y_{0.875}Zr_{0.1}Ce_{0.025}$ $(BO_3)_3$ (ZC-LYBO)

$Li_{6-x}Y_{1-x}Zr_x(BO_3)_3$	*relative density* / %
x = 0	77.1
x = 0.025	88.2
x = 0.05	95.2
x = 0.075	95.9
x = 0.1	88.2
x = 0.2	93.0
x = 0.3	90.8
x = 0.4	91.1
x = 0.6	95.2
$Li_{6-y}Y_{1-y}Ce_y(BO_3)_3$	*relative density* / %
y = 0.025	89.3
$Li_{6-x-y}Y_{1-x-y}Zr_xCe_y(BO_3)_3$	*relative density* / %
x = 0.1, y = 0.025	88.9

Table S3 Ionic conductivities for undoped $Li_6Y(BO_3)_3$, Zr-doped $Li_{6-x}Y_{1-x}Zr_x(BO_3)_3$, Ce-doped $Li_{5.975}Y_{0.975}Ce_{0.025}(BO_3)_3$, and Zr,Ce-doped $Li_{5.875}Y_{0.875}Zr_{0.1}Ce_{0.025}$ $(BO_3)_3$ (ZC-LYBO) at 27 °C

$Li_{6-x}Y_{1-x}Zr_x(BO_3)_3$	σ / S cm^{-1} at 27 ^{o}C
x = 0	5.6×10^{-11}
x = 0.025	5.8×10^{-6}
x = 0.05	8.8×10^{-6}
x = 0.075	1.3×10^{-5}
x = 0.1	1.4×10^{-5}
x = 0.15	9.5×10^{-6}
x = 0.2	1.0×10^{-5}
x = 0.3	6.7×10^{-6}
x = 0.4	4.7×10^{-6}
x = 0.6	5.5×10^{-7}
$Li_{6-y}Y_{1-y}Ce_y(BO_3)_3$	σ / S cm^{-1} at 27 ^{o}C
y = 0.025	6.9×10^{-7}
$Li_{6-x-y}Y_{1-x-y}Zr_xCe_y(BO_3)_3$	σ / S cm^{-1} at 27 ^{o}C
x = 0.1, y = 0.025	1.7×10^{-5}

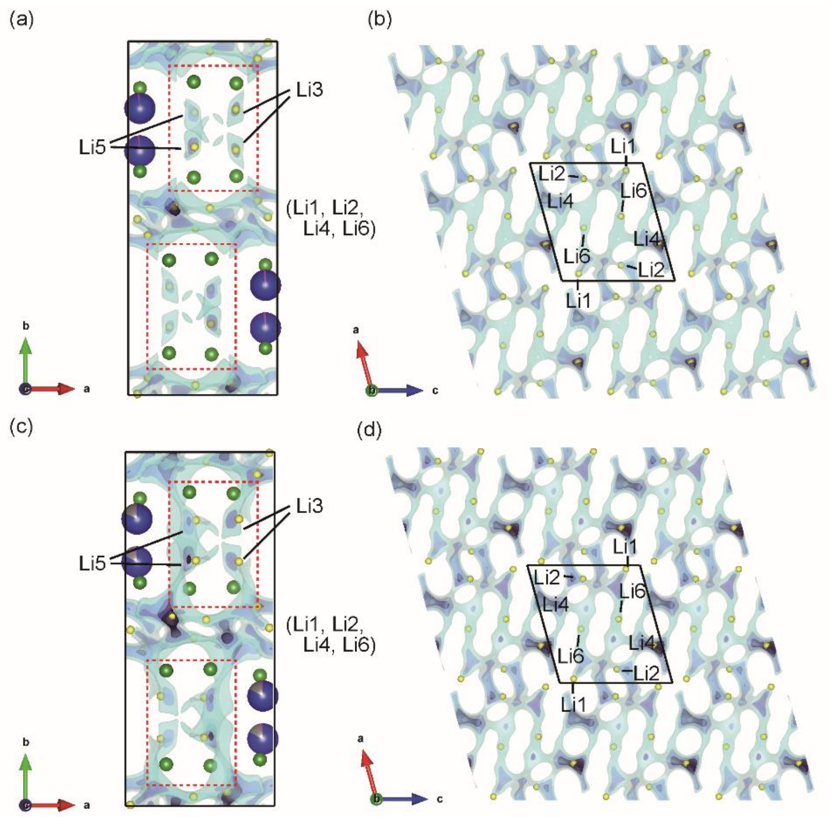

Fig. S4 3D BVSE maps of (a) the *ab* plane and (b) *ac* plane for Ce-doped $Li_{5.975}Y_{0.975}Ce_{0.025}(BO_3)_3$, and of (c) the *ac* plane and (d) *ab* plane for Zr,Ce-doped $Li_{5.875}Y_{0.875}Zr_{0.1}Ce_{0.025}$ $(BO_3)_3$ (ZC-LYBO).

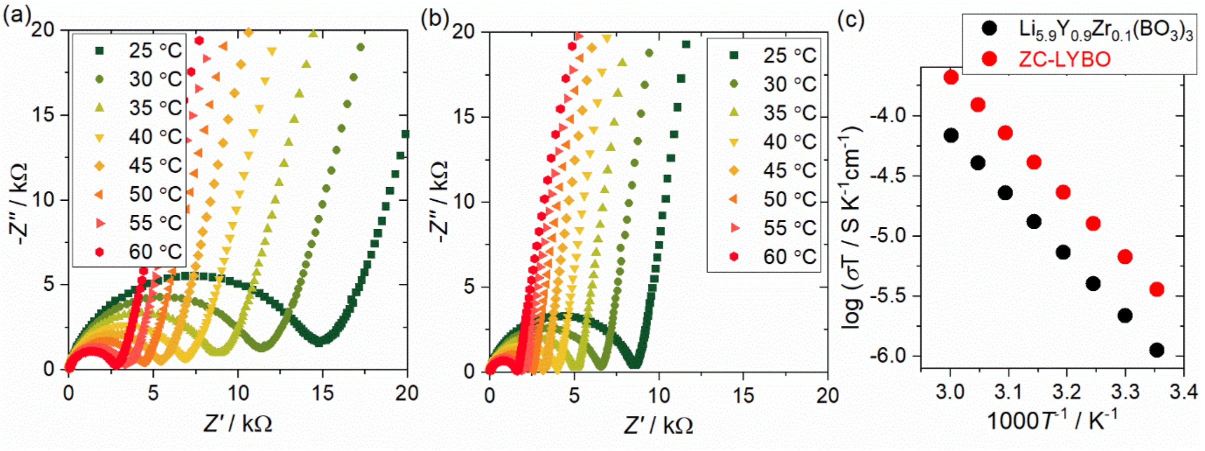

Fig. S5 Nyquist plots for (a) Zr-doped $Li_{5.9}Y_{0.9}Zr_{0.1}(BO_3)_3$ and (b) Zr,Ce-doped $Li_{5.875}Y_{0.875}Ce_{0.025}Zr_{0.1}(BO_3)_3$ (ZC-LYBO) at various temperatures. (c) Arrhenius plots of Zr-doped $Li_{5.9}Y_{0.9}Zr_{0.1}(BO_3)_3$ (black) and Zr,Ce-doped $Li_{5.875}Y_{0.875}Zr_{0.1}Ce_{0.025}$ $(BO_3)_3$ (ZC-LYBO) (red).
